# Diabetic retinopathy: a complex pathophysiology requiring novel therapeutic strategies

**DOI:** 10.1080/14712598.2018.1545836

**Published:** 2018-11-14

**Authors:** Michael Whitehead, Sanjeewa Wickremasinghe, Andrew Osborne, Peter Van Wijngaarden, Keith R. Martin

**Affiliations:** a John van Geest Centre for Brain Repair, Department of Clinical Neurosciences, University of Cambridge, Cambridge, UK; b Centre for Eye Research Australia, University of Melbourne and Royal Victorian Eye and Ear Hospital, Melbourne, Australia; c Department of Surgery, University of Melbourne, Melbourne, Australia; d Eye Department, Addenbrooke’s Hospital, Cambridge, UK; e Cambridge NIHR Biomedical Research Centre, Cambridge, UK; f Wellcome Trust – MRC Cambridge Stem Cell Institute, University of Cambridge, Cambridge, UK

**Keywords:** Diabetic retinopathy, diabetic macular edema, neovascularization, vascular endothelial growth factor, neuronal apoptosis

## Abstract

**Introduction**: Diabetic retinopathy (DR) is the leading cause of vision loss in the working age population of the developed world. DR encompasses a complex pathology, and one that is reflected in the variety of currently available treatments, which include laser photocoagulation, glucocorticoids, vitrectomy and agents which neutralize vascular endothelial growth factor (VEGF). Whilst these options demonstrate modest clinical benefits, none is yet to fully attenuate clinical progression or reverse damage to the retina.

This has led to an interest in developing novel therapies for the condition, such as mediators of angiopoietin signaling axes, immunosuppressants, nonsteroidal anti-inflammatory drugs (NSAIDs), oxidative stress inhibitors and vitriol viscosity inhibitors. Further, preclinical research suggests that gene therapy treatment for DR could provide significant benefits over existing treatments options.

**Areas covered**: Here we review the pathophysiology of DR and provide an overview of currently available treatments. We then outline recent advances made towards improved patient outcomes and highlight the potential of the gene therapy paradigm to revolutionize DR management.

**Expert opinion**: Whilst significant progress has been made towards our understanding of DR, further research is required to enable the development of a detailed spatiotemporal model of the disease. In addition, we hope that improvements in our knowledge of the condition facilitate therapeutic innovations that continue to address unmet medical need and improve patient outcomes, with a focus on the development of targeted medicines.

## 1. Introduction

Diabetic retinopathy (DR) is the leading cause of vision loss in the working age population of the developed world [1]. Clinically, DR is characterized by retinal neovascularization, the formation of microaneurysms, the presence of protein exudates in the vitreous, and ultimately a steady decline in visual acuity in patients, of which around 13 million are thought to exist in developed countries [1].

The pathophysiology of DR is driven by prolonged hyperglycemic episodes (elevated blood glucose concentrations) arising from suboptimal glycemic control in patients with either type I or II diabetes mellitus (DM) using dietary modifications, oral drug therapy (metformin, sulphonylurea, dipeptidyl-peptidase-4 inhibitors, sodium-glucose cotransporter-2 inhibitors), or injected insulin formulations. In patients, elevated blood glucose levels drive aberrant regulation of a number of biochemical pathways, ultimately leading to superoxide production and the burden of oxidative stress in retinal tissues. Mitochondrial dysfunction, inflammation, and hypoxia-driven vascular endothelial growth factor (VEGF) secretion accordingly give rise to vascular and neuronal apoptosis, and neovascularization and elevated vasopermeability, respectively [2–4].

The complex pathology of DR reflects the variety of currently available treatments, which include laser photocoagulation, glucocorticoids, vitrectomy, and agents that neutralize VEGF. Whilst these options demonstrate modest clinical benefits, none is yet to fully attenuate clinical progression or reverse damage to the retina. In addition, many require frequent administration involving intraocular injections, which may be associated with side effects such as corneal scarring, not to mention the costs associated with frequent ophthalmology clinician visits [3,5].

This has led to an interest in developing novel therapies for the condition, such as mediators of angiopoietin signaling axes, immunosuppressants, nonsteroidal anti-inflammatory drugs (NSAIDs), oxidative stress inhibitors, and vitriol viscosity inhibitors (VVIs). Further, preclinical research suggests that gene therapy treatment for DR could provide significant benefits over existing treatments options [6].

Viral vector-based systems have been utilized for a number of inherited orphan ophthalmic conditions involving single-gene mutations (e.g. choroideremia, Leber’s congenital amaurosis, Leber’s hereditary optic neuropathy) and recent research suggest that gene therapy treatment for DR could translate well to patients through a one-time therapeutic drug administration that has the ability to selectively target diseased areas of the retina [7].

## 2. Clinical presentation and diagnosis

DR is the commonest cause of vision loss in adults aged 20–74 years [1]. An estimated 285 million people suffer from diabetes, and one-third of these are affected by vision-threatening DR, which may include diabetic macular edema (DMO) or proliferative DR (pDR) [8].

In patients with type I diabetes, pDR is the most prevalent vision-threatening condition. In type II diabetics however, DMO is more common, and this explains the significant increase seen with the prevalence of DMO over recent years, in which everincreasing levels of obesity in the western world have been implicated [1].

Clinically, pDR and DMO may present with a variety of symptomatic ailments and variability between patients is common. During the early stages of the disease, patients are often asymptomatic but progress over time to develop microaneurysms, hemorrhages, and intraretinal microvascular abnormalities [9]. Upon examination, this can manifest as dark spots occluding vision, blurred vision, impaired color vision, and eventually vision loss if treatment is not effective [10]. As DR progresses, DMO may occur, defined as the presence of retinal thickening and hard exudates within 500 µm of the center of the macula [11]. Additionally, some patients demonstrate severe pDR in which aberrant neovascularization leads to the formation of highly permeable blood vessels across the retina.

To diagnose DR, ophthalmologists use fluorescein angiography and optical coherence tomography (OCT) to assess retinal blood vessel permeability (leakage) and thickness, respectively [12]. Newer techniques, including OCT angiography, are also becoming more widely used.

## 3. Clinical pathophysiology

In DR, blood–retinal barrier (BRB) dysfunction is common. The leakage of blood constituents into the retinal neuropile occurs in conjunction with the degradation of the inner BRB, and this often leads to DMO. VEGF is strongly implicated in the leakage of blood vessels and acts as a potent vasopermeability agent [2]. Thickening of the vascular basement membrane is also common in DR and is thought to arise from the upregulation of fibronectin, collagen, and laminin, which in turn leads to changes in the microenvironmental factors mediating the growth, survival, and function of pericytes and endothelial cells [13].

Central to the degenerative capillary pathophysiology seen in DR is the loss of pericyte function. Pericytes play a crucial role in normal retinal function, facilitating the differentiation, migration, and proliferation of angiogenic endothelial cells [14]. The loss of pericytes and the presence of ‘pericyte ghosts’ are therefore considered a key histopathological hallmark of DR [15].

Microaneurysms are seen early in the development of DR and are often the first clinically recognizable features. These present as ‘balloon-like’ protrusions of the capillary wall and are known to recruit inflammatory cells which further damage the endothelial lining. Herein, late-stage microaneurysms are sometimes sclerotic and frequently exist in the absence of an endothelial lining and are associated with regions of extensive capillary degeneration [16].

## 4. Molecular pathophysiology

**Figure 1.**
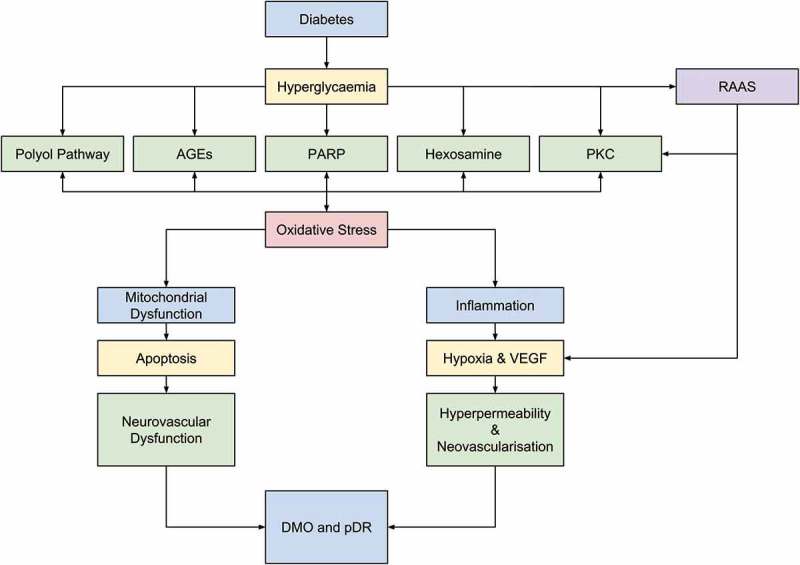
Diabetes leads to hyperglycemic episodes which in turn impacts five key biochemical pathways: – polyol pathway activation; production of advanced glycation endproducts (AGEs); protein kinase C (PKC) activation; hexosamine pathway activation; and poly (ADP-ribose) polymerase upregulation. This in turn leads to oxidative stresses, resulting in mitochondrial dysfunction, deregulation of proinflammatory mediators and crucially, hypoxia. These effects cause apoptosis of vascular and neuronal cells and upregulation of VEGF expression, eventually leading to neurovascular dysregulation, and hyperpermeable blood vessels and/or neovascularization. Importantly, the generation of ROS and oxidative stress further exacerbates metabolic dysfunction, itself leading to elevated ROS production in a self-perpetuating positive feedback mechanism. In addition, the renin angiotensin aldosterone system is implicated in driving neurovascular dysfunction. Reproduced from Pharmacology & Therapeutics, Vol 173, Wang et al., Gene therapy for diabetic retinopathy: Are we ready to make the leap from bench to bedside?, Copyright 2017, with permission from Elsevier [7].

The biology of DR is hugely complex, and many of the underlying mechanisms of the disease are yet to be fully understood. Figure 1 summarizes key diabetic-related factors implicated in the development of DR. Poor DR management leads to hyperglycemic episodes which drive metabolic dysfunction, resulting in oxidative stress and the generation of reactive oxygen species (ROS, e.g. superoxide-free radicals). Mitochondrial aberrations may induce apoptosis and inflammatory factors may elevate hypoxia-mediated VEGF secretion. Neurovascular dysfunction, vascular hyperpermeability, and/or neovascularization may follow and give rise to the pathological hallmarks of pDR and DMO [7].

### 4.1. The polyol pathway

Hyperglycemia associated with poor DR management drives the aberrant regulation of five biochemical pathways in DR patients, resulting in excess glucose being metabolized via the polyol pathway through an aldose reductase (AR)-mediated pathway which produces sorbitol [17]. Because sorbitol is impermeable to cellular membranes, it accumulates inside the cell and induces osmotic damage, amongst other harmful effects [18,19]. Sorbitol can also be metabolized to fructose via a sorbitol dehydrogenase-mediated pathway, and subsequently to fructose-3-phosphate and deoxyglucosone, both of which are strong glycolyzing agents and lead to the deposition of advanced glycation endproducts (AGEs) [17].

In addition, upregulation of the polyol pathway results in a reduction in the availability of NADPH, thereby enhancing the sensitivity of affected cells to oxidative stress and also depleting the reduced glutathione pool, itself leading to elevated ROS levels. Recent evidence also suggests that the sorbitol dehydrogenase-mediated shift in NADH/NAD+ levels may also be further exacerbated by NAD+-depleting NADH oxidase downregulation, itself leading to increased levels of ROS within cells, and also the inhibition of NAD+-dependent glyceraldehyde phosphate dehydrogenase (GAPDH) enzymatic activity. As discussed later, reductions in GAPDH activity have been implicated as a causative biochemical pathway in DR [20,21].

### 4.2. AGEs formation

The formation and accumulation of AGEs is markedly increased in diabetic patients in accordance with the enhanced availability of glucose [22,23]. One key pathological property of AGEs is their capacity to cross-link proteins, thereby altering their structure and function, and in diabetes, this includes basement membranes, cellular receptors, and blood vessel wall components. Moreover, AGEs can activate their cognate receptors to induce prooxidant and pro-inflammatory events, thus exacerbating oxidative stress and leukocyte adhesion in DR sufferers [24].

These mechanisms have now been linked to the molecular pathophysiology of DR. The accumulation of AGEs has been correlated to pericyte loss, whilst treatment of diabetic rats with aminoguanidine hydrochloride – an AGE formation inhibitor – limited microaneurysm and acellular capillary formation [25]. Similarly, treatment with a vitamin B6 derivative and AGE formation inhibitor, pyridoxamine, protected against capillary dropout and also downregulated the expression of basement membrane components [26].

### 4.3. Protein kinase C activation

Hyperglycemic episodes also lead to increased glucose flux via the glycolysis pathway. This elevates synthesis of diacylglycerol (DAG) which in turn activates the protein kinase C (PKC) pathway [27]. PKC mediates a plethora of biochemical signaling pathways, and as a result, it impacts a number of molecular processes when upregulated in DR, including activation of the mitogen-activated protein kinase (MAPK) factors, themselves leading to enhanced expression of stress-related proteins like c-Jun kinases and heat shock proteins, two key mediators of vascular function [28].

In particular, the PKC-β isoform has been shown to drive VEGF expression, a key regulator of vascular permeability and angiogenesis underpinning the molecular pathophysiology of DR [29].

PKC activation also drives overexpression of NADPH oxidase and NFκB in a number of vascular cells – including endothelial cells, smooth muscle cells, and pericytes – thereby exacerbating the oxidative stresses and inflammatory pathophysiological moieties associated with DR [30].

### 4.4. Hexosamine pathway flux

In the hexosamine pathway, fructose-6-phosphate (F6P) is converted into *N*-acetylglucosamine-6-phosphate (GlcNAc) by glutamine F6P amidotransferase. GlcNAc is then converted into uridine-5-diphospho-*N*-acetylgalactosamine (UDP-GlcNAc), a key regulator of a huge number of cytoplasmic and nuclear proteins [31]. In DR, SP1 transcription factor-dependent increases in transforming growth factor beta (TGFβ) and plasminogen activator inhibitor-1 (PAI-1) expression have been suggested to occur in smooth muscle cells, glomerular mesangial cells, and aortic endothelial cells [32–34].

*O*-GlcNAc transferase (OGT) catalyzes the addition of GlcNAc to serine and threonine residues at phosphorylation sites on SP1, thereby upregulating its transcriptional activity and pathological TGFβ and PAI-1 expression [35]. In addition, the glycosylation of RNA polymerase-II transcription factors by OGT and UDP-GlcNAc has been proposed as a more generalized mechanism by which glucose-responsive gene transcription is regulated, in turn leading to the dysregulation of the expression of a number of proteins that ultimately lead to DR pathophysiology [36].

### 4.5. Poly(ADP-ribose) polymerase activation

Hyperglycemia-induced oxidative stress has been shown to correlate to increased poly(ADP)-ribose polymerase (PARP) activation, via a ROS-mediated DNA damage-dependent mechanism. This in turn leads to NAD+ depletion and concomitant inhibition of GAPDH through the depletion of the enzyme’s catalytic cofactor and PARP-mediated ribosylation. In conjunction, these molecular mechanisms have been shown to contribute to endothelial cell dysfunction in diabetic blood vessels in various diabetic complications including DR. Moreover, the inhibition of PARP has demonstrated protection against diabetes-induced retinopathy, thereby substantiating the role of this pathway in the DR pathology [37,38].

### 4.6. Renin–angiotensin aldosterone system activation

The renin–angiotensin aldosterone system (RAAS) is an endocrine system that regulates systemic blood pressure. Although the exact mechanism that RAAS plays within DR is yet to be elucidated, its role as a regulator of vascular hydrodynamics and findings that purport the upregulation of RAAS components in DR patients implicates it in the onset and progression of the condition.

Activation of the RAAS in DR begins with the local accumulation of glucose and its metabolite, succinate. This in turn leads to the activation of GPR91, a G-protein-coupled receptor, that stimulates juxtaglomerular cells to release prorenin and renin. Further, expression of angiotensin-converting enzyme (ACE) in the retina has been reported to adversely affect capillary perfusion and vascular structure. Herein, ACE-mediated VEGF upregulation has been reported and correlated to elevated progression of DR [39]. These findings have been corroborated by in-human clinical evidence showing that lisinopril-mediated ACE inhibition can prevent the formation of new blood vessels in the diabetic eye [40]. In support of this, another study has demonstrated that losartan-mediated inhibition of the angiotensin-II type I receptor prevented neovascularization in diabetic subjects [1,41].

### 4.7. Oxidative stresses and superoxide production

Throughout this discussion of the pathology of DR, frequent reference has been made to the role of oxidative stress. Indeed, oxidative stress (e.g. production of superoxide) has been proposed as a ‘unifying mechanism’ of DR that acts as a common element linking all of the hyperglycemia-induced biochemical and molecular pathways. This has primarily been driven by evidence that all of the pathogenic mechanisms outlined above drive the production of superoxide by the electron transport chain [42,43].

The mediation of these pathways by mitochondrial superoxide is dependent on the capacity of superoxide ions to inhibit GAPDH activity, and 66% superoxide-mediated reductions in GAPDH activity have been measured [42]. As described previously, ROS-mediated GAPDH inhibition is likely an indirect effect and involves the activation of PARP and concomitant depletion of NAD+ and GAPDH ribosylation, as opposed to the direct inhibition of GAPDH by ROS [44].

**Figure 2.**
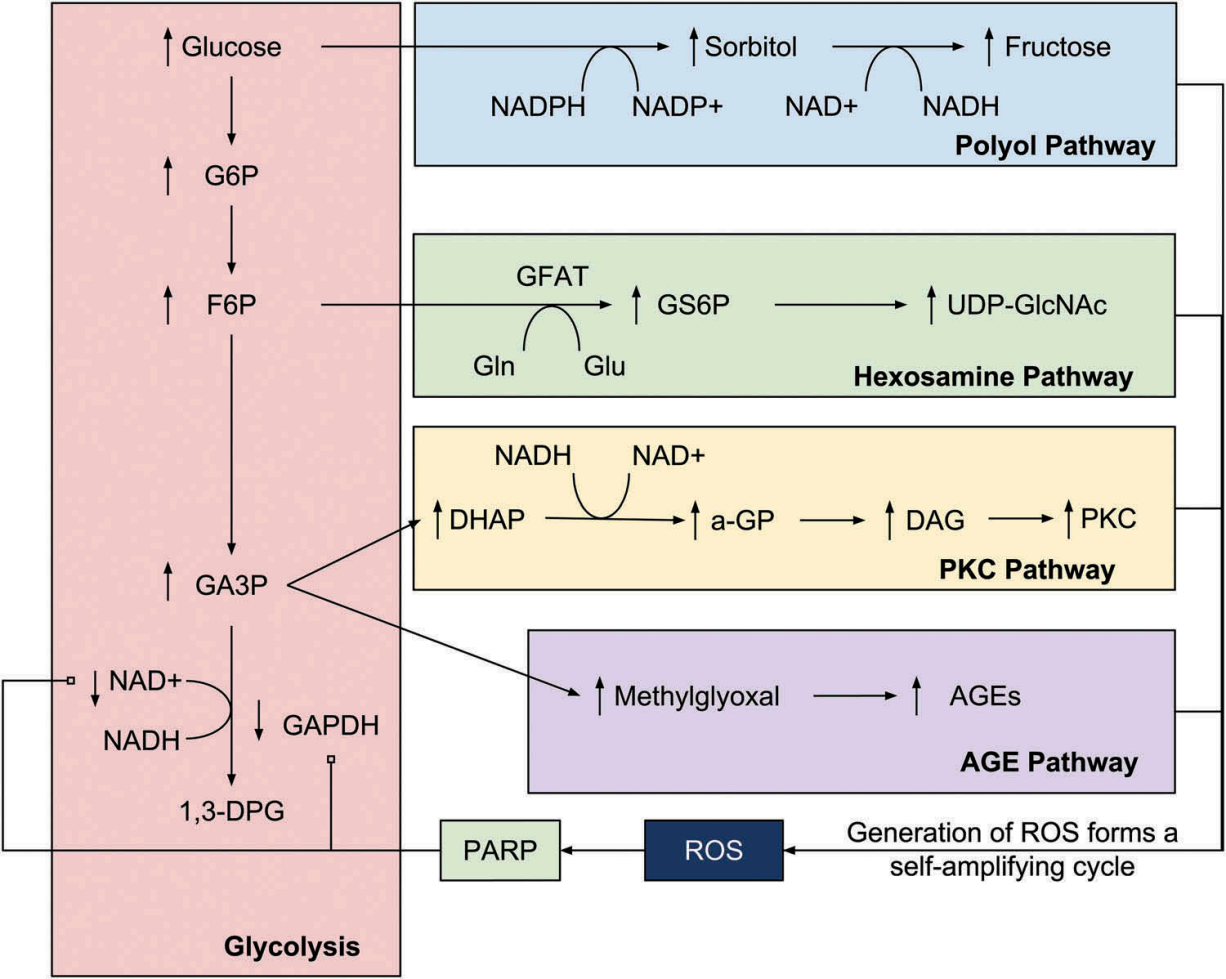
The relationship between superoxide and ROS production and the key pathologic pathways of DR. This model demonstrates the centrality of oxidative stress to DR and has led some to purport superoxide production to be the ‘unifying mechanism’ in the complex pathology of DR. G6P = glucose-6-phosphate, F6P = fructose-6-phosphate, GA3P = glyceraldehyde-3-phosphate, 1,3-DPG = 1,3,-diphosphoglycerate, GS6P = glucosamine-6-phosphate, a-GP = alpha-glycerol-phosphate. Reproduced with permission from Springer Nature, Copyright 2001 [45].

As outlined in Figure 2, superoxide causes an elevation in the levels of glyceraldehyde-3-phosphate (G3P) by inhibiting its NAD+-dependent conversion to 1,3-diphosphoglycerate via the inhibition of GAPDH activity. G3P in turn upregulates the formation and deposition of AGEs by accelerating the addition of triose phosphates to methyl-glyoxal, the main AGE precursor. This hypothesis is supported by data stating that the antisense oligonucleotide-mediated inhibition of GAPDH enhances the rate of addition of triose phosphates and AGE formation [42].

G3P also upregulates the PKC pathway by enhancing the conversion of dihydrooxyacetone phosphate to DAG, a key activator of PKC, and this effect has been corroborated by data showing that the inhibition of GAPDH by antisense oligonucleotides has a similar effect [45]. G3P upregulation increases the availability of F6P which in turn drives flux through the hexosamine pathway through the enhancement of glucosamine-6-phosphate and ultimately UDP-GlcNAc levels. Finally, G3P upregulation enhances the flux through the polyol pathway by increasing the availability of glucose [45]. Therefore, the role of ROS in mediating the four metabolic pathways described here constitutes a self-amplifying cycle in which the generation of superoxide drives metabolic dysfunction, which in turn drives more ROS production via a self-perpetuating positive feedback mechanism.

### 4.8. Inflammation

The role of inflammation and leukostasis in DR is now well documented and is a key driver of capillary occlusion and hypoxia that ultimately drives VEGF expression and concomitant hallmark vascular abnormalities that characterize DR. In DR patients, a significant increase in systemic pro-inflammatory cytokine expression is seen and the elevation of chemokine synthesis in the retina is also present. Several studies have also reported that the relative expression of these factors is correlated to the rate of progression of DR and recent evidence has also implicated the activation of various immune cells with the onset of the condition [46–48].

The central role of inflammation in driving VEGF secretion and thereby the pathology of DR is demonstrated by a number of studies on the effects of inflammation inhibition preclinically and in human clinical trials [49,50]. The use of anti-inflammatory drugs such as intravitreal triamcinolone acetonide (IVTA) and NSAIDs like nepafenac has been shown to reduce VEGF expression, reduce vascular permeability, inhibit retinal cell death, diminish leukostasis, and ultimately improve visual acuity [49–51].

### 4.9. Hypoxia and VEGF

Perhaps the best-characterized growth factor involved in the development of DR is VEGF. The role of VEGF in promoting angiogenesis, the breakdown of the BRB, and vascular hyperpermeability are now well described in the development and progression of both pDR and DMO [52–54]. VEGF can exist as one of four isoforms, VEGF-121, -165, -189, or -206, and of these VEGF-165 has been the most heavily implicated in the DR pathology [55,56]. VEGF exerts its function on cells through its binding to and activation of two membrane-bound tyrosine kinase receptors, VEGF-R1 (FLT1) and VEGF-R2 (FLK1), on endothelial cells, of which VEGF-R2 appears to be the primary mediator of VEGF activity. VEGF binding to its cognate receptor activates calcium influx channels or MAPK signaling pathways in order to mediate its physiological effect [57,58].

### 4.10. Mitochondrial dysfunction

In contrast to that of inflammation and VEGF upregulation, the role of mitochondrial dysfunction in DR is poorly understood. A number of hypotheses have been suggested, however, to explain how superoxide ions and other ROS lead to aberrations in mitochondrial function. One paradigm is that the hyperglycemia-derived ROS and concomitant oxidative stresses detailed above lead to compromised function of the electron transport chain, itself leading to damages to mitochondrial DNA [3,4,59,60].

### 4.11. Vascular and neural cell apoptosis

The loss of vascular cells, visualized as acellular capillaries, is also well characterized in DR. Terminal dUTP nick-end labeling (TUNEL) has been applied to human and animal model samples and demonstrated the elevation of this apoptotic marker in DR retinas compared to normal controls [61]. In particular, the appearance of ‘pericyte ghosts’ has been related to apoptotic casualties, in which upregulation of the BCL2 family member BAX has been implicated.

The onset of neural cell apoptosis, however, is less well documented but has still been proposed by some to represent a novel paradigm in our understanding of the pathology and treatment of DR [62,63]. In the inner retinal layer in particular, TUNEL analysis has demonstrated elevated apoptosis of retinal ganglion cells (RGCs). The onset of neuronal apoptosis has also been shown to proceed vascular apoptosis, perhaps highlighting the sensitivity of neuronal cell types to the apoptotic stresses outlined earlier, those principally being oxidative stress and concomitant mitochondrial dysfunction [64–66].

### 4.12. The neurovascular unit

The term ‘neurovascular unit’ (NVU) refers to the coupling of neuronal and vascular cell types in the retina and incorporates RGCs, bipolar cells, horizontal cells, astrocytes, Muller cells, microglia, pericytes, and endothelial cells. The NVU regulates the flow of blood throughout the inner retina via an autonomic innervation-independent mechanism, and instead, it responds to neural and circulatory cues in order to regulate blood flow [67,68].

In DR, normal retinal blood flow is disrupted and there are now substantial amounts of evidence to suggest that the function of the NVU is also affected by the condition. In particular, responses to hyperemia are diminished and this can be demonstrated in a clinical setting using flicker-evoked vasodilation in patients asymptomatic for DR, suggesting that NVU uncoupling may be a key causative factor in the disease [69].

## 5. Current treatment paradigms

### 5.1. Glycemic control

A number of large clinical trials (DCCT, UKPDS, and ACCORD) have demonstrated that the onset and severity of DR can be prevented using insulin injections. Typically, this involves reducing blood-glucose levels as much as possible whilst avoiding the onset of severe side effects. This has proven to be a largely effective technique with one trial, the Epidemiology of Diabetes Interventions and Complications study, demonstrating that the positive effects of glycemic control can extend up to 10 years. However, these benefits can reduce slightly over time and the ‘metabolic memory’ phenomenon can also mitigate the impact of insulin in patients [70–72].

### 5.2. Laser photocoagulation

In a US-based, large-scale, randomized clinical trial, macular laser treatment has been shown to reduce the risk of visual loss in DMO patients from 28% to 11%. Further, studies seeking to compare the benefits of photocoagulation and scatter laser treatment have demonstrated superiority of the focal photocoagulation technique at inhibiting moderate vision loss with no deleterious effects on visual fields [73,74].

### 5.3. RAAS blockade

One study sought to compare the effects of the ACE inhibitor enalapril with the angiotensin-II receptor type I inhibitor losartan on the progression of DR. No statistically significant effect was found, suggesting that either mechanism of inhibiting the RAAS pathway was equally effective. In addition, the use of candesartan cilexetil, an angiotensin-II receptor inhibitor, in DR-naive and mild DR resulting from DM type I was shown to inhibit the progression of the condition [75–77].

### 5.4. Glucocorticoid therapy

The use of systemic and ocular glucocorticoid treatments, including IVTA (a long-acting nonsoluble hormone), for a number of eye treatments, has been part of clinical practice for over 50 years. IVTA has been shown to reduce macular edema in DMO and wet age-related macular degeneration (wAMD). IVTA injections demonstrated reductions in retinal thickness and concomitantly improvement in 56% of eyes by five lines in the Snellen eye chart in a 2003 clinical trial [54,78,79].

### 5.5. PKC inhibitors

Ruboxistaurin, a PKC-beta-specific inhibitor, has been shown to inhibit diabetic BRB breakdown and retinal neuropathy in animal models of DR. In human clinical trials however, modest reductions in vision loss were seen in the study group, and the effect of the drug was only significant when combined with laser photocoagulation [80].

### 5.6. Fenofibrate

Fenofibrate is a peroxisome proliferator-activated receptor alpha activator that increases lipolysis and the elimination of triglycerides from the blood plasma via the upregulation of lipoprotein lipase and the downregulation of apoprotein C III synthesis. Whilst recent evidence suggests that the risk of DR onset and development is mitigated with fenofibrate treatment in patients with DM type II, the mechanism by which this occurs appears to act independently of the lowering of systemic high-density lipid and mean triglyceride levels in the FIELD and ACCORD clinical trials [81,82].

### 5.7. Anti-VEGF therapeutics

The inhibition of VEGF has been a cornerstone of the treatment of DMO and wAMD for over 10 years, and the benefits of anti-VEGF therapy have been well documented in a number of phase III clinical trials for retinal diseases. The first FDA approval of a monoclonal antibody therapeutic for DMO was Lucentis (ranibizumab, Roche Genentech) in 2006, and since then, the molecular principles of antibody-based target inhibition in DMO have been built upon through the development of single chain variable fragments and other derivatives of the monoclonal antibody paradigm.

More recently, a new generation of anti-VEGF molecules have been developed by Regeneron Inc. Aflibercept, a so-called VEGF trap, is a 115-kDa fusion protein exhibiting high affinity for VEGF-RI and -RII antagonists. In phase III clinical development, aflibercept has demonstrated potent antitumor neovascularization capabilities in colorectal cancer patients, a finding corroborated by recent reports of efficacy in phase III trials for retinal vein occlusion-induced macular edema and DMO [83].

### 5.8. Limitations and drawbacks to current therapies

Whilst a number of therapeutic strategies are available to clinicians for the treatment of DR, no treatment is yet to fully attenuate clinical progression to reverse damage to the retina. Further, a large number of patients do not respond to certain treatments at all, and patient outcomes are often limited in many cases. A summary of the limitations and drawbacks to existing therapies is given in Table 1.

**Table 1.** An overview of the limitations and drawbacks associated with the treatment strategies currently utilized for DR management.

Treatment	Pathway	Limitations and drawbacks
Glycemic control	Insulin signaling	Worsening of symptoms seen in patients over time Side effects can include headaches, weight gain, rashes, and inflammation at the site of injection [84] Metabolic memory phenomenon limits efficacy in poorly managed cases of DM [85]
Laser photocoagulation	Macular edema	Can result in scarring of the retina and apoptosis of retinal pigment epithelium and other retinal cell types, reducing visual acuity Choroidal neovascular membranes can develop if the laser scar affects the Bruch’s membrane [86]
Enalapril, losartan	RAAS	Significant renal and cardiovascular side effects seen in some patients, including hyperkalemia and worsening renal function [87]
Triamcinolone acetonide	Glucocorticoid signaling	Secondary ocular hypertension (40%), elevated intraocular pressure (2%), and nuclear cataracts (20%) are common side effects [88]
Ruboxistaurin	PKCβ signaling	Modest clinical benefits and only statistically significant improvements seen when combined with laser photocoagulation [89]
Fenofibrate	PPARɑ activator	Substantial side effect profile which includes stomach pain, nausea and vomiting, and muscle pain 20-h half-life necessitates daily-dosing regimen [90]
Bevacizumab, ranibizumab, aflibercept	VEGF signaling	High prevalence of nonresponders Resistance to therapy seen with repeated administration Repeated intravitreal injections has detrimental impact, including corneal scarring [91]

RAAS: Renin–angiotensin aldosterone system; PKC: protein kinase C; PPAR: Peroxisome proliferator activator protein; VEGF: vascular endothelial growth factor.

## 6. Recent therapeutic developments

As detailed above, although a number of treatments are now approved for DR, each is beset with its own limitations and drawbacks. Therefore, a number of alternative pathways have been considered as novel therapeutic options for the condition.

### 6.1. Angiopoietin and Tie2 ligand/receptor interactions

The angiopoietin family is involved in the regulation of vascular maturation and neovascularization. Angiopoietin-1 and -2 (Ang1 and 2) are known to have opposing effects when binding to their cognate receptor, the receptor tyrosine kinase Tie2. Ang1 induces Tie2 activation and phosphorylation, thereby promoting vessel stabilization and maturation. In addition, Ang1 has demonstrated anti-inflammatory effects and the ability to inhibit leukocyte adhesion to endothelial cells in animal models [92,93]. By contrast, Ang2 is a context-dependent mediator of the Tie2 pathway and can act as a mild agonist or an antagonist of the receptor. To dephosphorylate and thereby deactivate the Tie2 receptor, Ang2 activates protein tyrosine phosphatase beta or vascular endothelial PTP, which in turn leads to blood vessel destabilization. This destabilization is essential for the normal process of angiogenesis but can lead to endothelial cell apoptosis in the absence of VEGF signaling [94,95].

The role of the angiopoietin family in DR has led to the instigation of a number of drug-development projects. Akebia Therapeutics have developed a number of Ang2 inhibitors with the aim of restoring Tie2 signaling, and Amgen has developed trebananib, a peptibody (peptide fused to an antibody Fc domain) targeting Ang1 and 2 proteins [96]. A similar approach has demonstrated clinical efficacy in phase II testing. Most recent attention has focused on RG7716, a bispecific antibody which can neutralize both VEGF and Ang2, and demonstrated superior efficacy against low-dose (0.3 mg) ranibizumab in recent US phase II clinical trials, perhaps highlighting the advantages of a combination-based approach to treating DR [97].

### 6.2. NSAIDs

As discussed above, the role of inflammation in driving the development of DR is now well characterized, and accordingly, clinical assessment of the use of NSAIDs has shown a positive effect at attenuating DR symptoms. One such NSAID is ketorolac, a prostaglandin synthesis inhibitor that targets the cyclooxygenase (COX) family of enzymes. In the clinic, intravitreal and topical administration of ketorolac has been shown to increase visual acuity via a reduction in inflammatory cytokine production [98–100]. In other assessments however, nepafenic, another COX inhibitor, was administered topically but failed to show any meaningful clinical benefit. On the whole, COX inhibitors are well tolerated in ophthalmic applications, although some serious side effects have been reported with ketorolac. In summary, NSAIDs represent a promising new therapeutic avenue for attenuating the impact of inflammation in the diabetic eye, whilst reducing the risk of cataracts and elevated intraocular pressure associated with the corticosteroids mentioned above [101–103].

### 6.3. Antibiotics and immunosuppressants

Several antibiotics have been shown to reduce the impact of inflammation in the diabetic eye in animal models and in-human clinical studies. Minocycline treatment, for example, has demonstrated reductions in cytokine levels in the retina in diabetic rats, a finding corroborated by clinical evidence showing decreased retinal thickness and vasopermeability and increased visual acuity following oral minocycline administration [104,105]. Interestingly, recent evidence purports that minocycline may in part function via the attenuation of the upregulation of PARP, thereby mitigating the impact of oxidative stresses and apoptotic stimuli in retinal tissues [106]. Another antibiotic and mediator of angiogenesis, squalamine, has demonstrated the capacity to reduce neovascularization in the diabetic eyes of multiple animal models of DR, and a clinical trial investigating the use of this drug in DM type I and type II has now been instigated in accordance [107,108].

Immunosuppressant drugs have achieved similar results in animal model and clinical studies. Sirolimus (also known as rapamycin) is known to have anti-angiogenic and antineoplastic attributes and decreases in VEGF synthesis and retinal cell proliferation that have been reported in sirolimus-treated cells from streptozotocin-challenged rat models of DR [109]. Both sirolimus and a similar immunosuppressant, everolimus, have demonstrated a reduction in neovascularization in mouse models of DR, a finding corroborated by clinical evidence reporting that bimonthly sirolimus injections decreased retinal thickness and improved visual acuity concomitantly in patients with DM type I and type II [110,111]. Mechanistically, these immunosuppressants are thought to exert their physiological effect via the inhibition of mammalian target of rapamycin kinase, an enzyme whose activity initiates pro-inflammatory and pro-angiogenic signaling cascades [112]. In summary, the promising results seen so far in the use of antibiotics and immunosuppressant drugs warrant further investigations, both preclinical and clinical, to further elucidate the potential of these classes of drugs for treating DR.

### 6.4. Targeting oxidative stresses

As outlined above, oxidative stress is thought to represent a ‘unifying mechanism’ in DR, wherein changes in the NADH/NAD+ ratio drive aberrant regulation of a number of biochemical pathways, mitochondrial dysfunction, and hypoxia-driven VEGF synthesis. In spite of the centrality of oxidative stress to the pathology of DR, conflicting evidence has arisen describing potential benefits that antioxidant use infers in patients. Whilst no association has been found between antioxidant usage and the incidence of DR in retrospective meta-analyses, one trial investigating DM type I and type II reported a preservation of visual function in the study group who consumed an antioxidant cocktail for six months [113,114].

One limitation of using conventional antioxidants is that they neutralize ROS with poor efficiency, whilst hyperglycemia-induced superoxide production occurs continuously. Superoxide dismutase (SOD) catalase mimetics demonstrate much greater ROS ablation efficiency, and treatment of DR animal models with these enzymes has been shown to abolish hyperglycemia-induced eNOS and prostacyclin synthase downregulation and concomitantly normalizes all five of the DR pathology-associated biochemical pathways [115,116].

Alternative approaches to targeting oxidative stress have seen more promising results however. Overexpression of uncoupling protein-1 (UCP-1) and manganese SOD (MnSOD) have been shown to reverse hyperglycemia-induced phenotypes in multiple cell types. In glomerular mesangial cells, MnSOD expression has been shown to attenuate hyperglycemia-induced collagen synthesis, and in dorsal root ganglion neurons (DRGs), MnSOD expression demonstrated a reduction in apoptosis. Further, overexpression of UCP-1 has been shown to inhibit apoptotic caspase cleavage and activation in rat DRGs, a finding corroborated by recent data purporting that UCP-1 or MnSOD expression in aortic endothelial cells inhibits macrophage adhesion and inflammatory activity, peroxisome proliferator-activated receptor gamma activation, and hyperglycemia-associated eNOS activity [45].

Similarly, the use of benfotiamine, a transketolase activator, has demonstrated the ability to attenuate hyperglycemia-mediated toxicity in endothelial cells, both *in vitro* and *in vivo* [117]. Mechanistically, benfotiamine functions by increasing the activity of the transketolase enzyme, thereby increasing the flux of F6P and G3P into pentose phosphates and diverting these metabolites away from the pathological biochemical pathways, outlined earlier in this article [118]. Clinically, benfotiamine has demonstrated the capacity to downregulate the damaging biochemical DR pathways in patients with DM type I, and further validation of the transketolase hypothesis has arisen from evidence reporting reduced albuminuria in DM type II treated with high doses of thiamine, the cofactor for transketolase enzymes [117,119,120].

PARP inhibitors have demonstrated the potential to reduce oxidative stresses in *in vitro* and *in vivo* models. In arterial endothelial cellular cultures, PARP inhibition was shown to attenuate concomitant activation of the PKC, AGE, and hexosamine pathways, and in animal models, PARP inhibition abolished vascular endothelial cell apoptosis [37,121].

Overall, these results warrant further investigation of oxidative stress inhibitors with the aim of developing clinical-stage candidates for the prevention and treatment of DR.

### 6.5. VVIs

Recent evidence has come to suggest that DMO patients with posterior vitreous detachment (PVD) exhibited lower rates of disease progression than patients without PVD. This suggests that intentional induction of PVD could be a viable therapeutic strategy, and several VVIs have been developed as a result. One such VVI is ocriplasmin (Thrombogenics), a protease delivered into the vitreous with a demonstrated capacity to reduce vitreal viscosity and vitreoretinal separation in animal models [122]. A recent phase III study investigating the impact of ocriplasmin in humans showed beneficial effects, leading to the FDA approval of the drug for vitreomacular adhesion [123,124]. These data suggest that the use of ocriplasmin could elicit positive effects in DR patients and warrants further investigation in diabetic models [123,124].

Similarly, luminate, an anti-integrin peptide, is another VVI currently in phase III clinical development for DMO. Integrins are well-characterized mediators of vitreoretinal adhesion and also regulate VEGF interactions with its cognate receptor, VEGF-RII (FLK1), and several integrin inhibitors have demonstrated the ability to reduce neovascularization in several DR animal models [125,126]. In accordance, luminate has been shown to target both vitreoretinal adhesion and angiogenesis, suggesting that it might be more effective than ocriplasmin when progressed into clinical-stage development [96].

### 6.6. Topical inhibitors of retinal neurodegeneration

Recent evidence has suggested that the topical administration of factors mediating the levels of glucagon-like peptide 1 (GLP1) in the diabetic retina may be a novel and promising therapeutic strategy for the condition. In a recent paper, topical administration of dipeptidyl peptidase IV inhibitors in mouse and human samples was able to prevent neurodegeneration and vascular leakage through a mechanism that involved the upregulation of GLP1 levels [127].

Further, the topical administration of a GLP1 receptor agonist in a diabetic mouse inhibited glial activation, neural apoptosis, and electroretinographical abnormalities, thereby preventing retinal neurodegeneration. This was shown to occur via the reduction in the levels of extracellular glutamate and concomitant elevation of pro-survival signaling pathway activation [127].

Taken in conjunction, these results suggest that mediation of the GLP1 signaling axis is a promising means of treating DR and also demonstrate the effectiveness of topical administration of therapeutic modalities for the condition.

## 7. Gene therapy for DR: challenges and opportunities

### 7.1. Gene therapy for ocular disorders

A number of pathways are being targeted as a means of developing novel therapeutic strategies for DR. Although no gene therapy clinical trials for DR have been undertaken to date, the eye is at the forefront of gene therapy research. Herein, the eye is relatively immune-privileged and only a few inflammatory events are associated with the introduction of viral particles. Various routes of administration are available, and this permits access to all tissue compartments that one could wish to target. Further, the anatomy of the eye is highly favorable, wherein the small size enables the use of low doses of vector for gene delivery and the use of fundus examination to enable ongoing assessment of treatment efficacy [7,128].

### 7.2. Targeting vasopermeability and neovascularization

As outlined earlier, a key mediator of neovascularization in DR is VEGF, and a number of VEGF-inhibiting gene therapies have now been described. These include attempts to ablate intraocular VEGF using sFLT1, a soluble splicing isoform of the VEGF-RI which acts as a decoy receptor for VEGF, thereby neutralizing the protein, and several reports have demonstrated the efficacy of this approach [129–132]. An overview of preclinical research seeking to target the vasopermeability and angiogenesis aspects of DR is given in Table 2. Across these therapeutic paradigms, the preference for the AAV2 vehicle is evident, as is the focus on the regulation of VEGF signaling.

**Table 2.** An overview of preclinical research seeking to address the vasopermeability and angiogenesis aspects of DR.

Reference	Vector type	Promoter	Transgene	Target
Tu et al. [133]	scAAV2	CMV	CAD180 and CAD112	Calrectulin signaling
Wu et al. [134]	AAV5	ICAM2	SpCas9 for VEGF-RII	VEGF signaling
Huang et al. [135]	AAV1	ICAM2	SpCas9 for VEGF-RII	VEGF signaling
Díaz-Lezama et al. [136]	AAV2	CMV	Vasoinhibin and sFlt-1	VEGF signaling
Biswal et al. [137]	scAAV2	GFAP	Endostatin	Endothelium
Haurigot et al. [138]	AAV2	CAG	PEDF	VEGF signaling
Pechan et al. [130]	AAV2	CMV	sFLT-1	VEGF signaling
Jiang et al. [139]	Lipofectamine	n/a	HIF1a and VEGF siRNA	HIF1a and VEGF signaling
Lamartina et al. [131]	Adenovirus	CMV/IRES-M2	sFLT-1	VEGF signaling
Ideno et al. [132]	AAV2/5	CMV	sFLT-1	VEGF signaling
Le Gat et al. [140]	Adenovirus	CMV	ATF, endostatin	uPA/uPAR signaling
Igarashi et al. [141]	Lentivirus	CAG	Angiostatin	Endothelium
Gehlbach et al. [129]	Adenovirus	CMV	sFLT-1	VEGF signaling
Auricchio et al. [142]	AAV1/2	CMV	PEDF, TIMP3, endostatin	Endothelium

ICAM2: Intracellular adhesion molecule 2; SpCas9: *Streptococcus pyogenes* Cas9 CRISPR system; scAAV2: self-complementary AAV2; GFAP: glial fibrillary acidic protein; sFLT-1: soluble FLT-1 (aflibercept); ATF: amino terminal fragment; uPA/uPAR: urokinase receptor; TIMP3: inhibitors of metalloproteinases; CAG: promoter sequence incorporating cytomegalovirus (CMV) enhancer elements and chicken β-actin promoter sequences; PEDF: pigment epithelium-derived factor; HIF1ɑ: hypoxia inducible factor alpha; ATF: activating transcription factor; IRES-M2: internal ribosome entry site. Adapted from Pharmacology & Therapeutics, Vol 173, Wang et al., Gene therapy for diabetic retinopathy: Are we ready to make the leap from bench to bedside?, Copyright 2017, with permission from Elsevier [7].

### 7.3. Preventing vascular and neuronal apoptosis

One attempt demonstrated that the introduction of soluble CD59, an inhibitor of the membrane attack complex known to contribute to apoptotic stimuli in DR [143], prevented disruption to the BRB and protected against damage to retinal neurons. In this study, the AAV-mediated delivery system was used in streptozotocin-induced diabetic models of DR, and a 60% reduction in vascular leakage from the retina was reported. Further, soluble CD59 was shown to activate retinal glial cells which are thought to protect RGCs from apoptotic stimuli [144].

An overview of preclinical research attempts seeking to address vascular and neuronal apoptosis in DR is given in Table 3. Again, AAV2 is the preferential vector serotype for retinal delivery, and a breadth of therapeutic targets is evident.

**Table 3.** An overview of preclinical research targeting vasodegeneration and neurodegeneration in DR.

Reference	Vector type	Promoter	Transgene	Target	Vascular protection	Neuronal protection
Dominguez et al. [145]	AAV	CBA	ACE2	RAAS system	Yes	n/a
Vacca et al. [146]	ShH10	CAG	Dp71	Muller cells	Yes	n/a
Zhang et al. [147]	AAV2	CAG	MnSOD	Superoxide	Yes	n/a
Xu et al. [148]	AAV2	CMV	EPO	EPO receptor	Yes	Yes
Hu et al. [149]	TransIT-TKO	U6	CTGF shRNA	CTGF	Yes	n/a
Adhi et al. [144]	AAV2/8	CAG	sCD59	MAC	Yes	Yes
Verma et al. [41]	AAV2	CAG	ACE2 or Ang(1–7)	RAAS	Yes	n/a
Gong et al. [150]	AAV	CBA	BDNF	Neuronal cells	n/a	Yes
Ramirez et al. [151]	AAV2	CAG	Vasoinhibin, PRL, sFLT-1	VEGF signaling	Yes	n/a
Shyong et al. [152]	AAV	CMV	Angiostatin	Endothelium	Yes	n/a

CAD180: Calrectulin anti-angiogenic domain; CAD112: CAD-like peptide 112; ShH10: a Muller cell-specific variant of the AAV vector; ACE2: angiotensin-converting enzyme 2; EPO: erythropoietin; CTGF: connective tissue growth factor; CAG: cytomegalovirus early enhancer; CBA: chicken-β actin promoter; MnSOD: manganese superoxide dismutase; U6: human RNA polymerase III promoter; MAC: membrane attack complex; BDNF: brain-derived neurotrophic factor; PRL: proteolytic cleavages of prolactin; ACE2: angiotensin-converting enzyme 2; sFLT-1: soluble FLT1 receptor (aflibercept). Adapted from Pharmacology & Therapeutics, Vol 173, Wang et al., Gene therapy for diabetic retinopathy: Are we ready to make the leap from bench to bedside?, Copyright 2017, with permission from Elsevier [7].

Overall, the gene therapy paradigm has shown promise as an alternative therapeutic strategy for the treatment of DR. The multitude of successes seen in preclinical animal model testing warrants further exploration of these treatments in in-human clinical testing. Ultimately, gene therapy could offer a one-off treatment for DR, which would mitigate the risk of corneal scarring associated with repeated intravitreal injections, and provide superior efficacy to the current standard-of-care, due to the constant therapeutic coverage that gene therapy-mediated drug delivery can offer.

## 8. Conclusion

The pathophysiology of DR is complex, with a large number of biochemical and molecular signaling pathways implicating in the onset and development of symptoms in patients. Here, the role of oxidative stress appears to be central, wherein the dysregulation of several biochemical pathways in DR induces superoxide production, itself driving further dysregulation of the molecular signaling pathways underpinning DR, in turn leading to more superoxide production. Also evident is the important role of vascular and neuronal apoptosis in the condition. Here, retinal degeneration in DR encompasses most of the cells found in the retinal microenvironment and suggests that future therapeutic strategies should target vascular and neuronal apoptotic pathways.

DR is a treatable condition, and a number of therapeutic options are available to ophthalmologists. Whilst these demonstrate modest clinical benefits, none is yet to fully attenuate disease progression or reverse damage to the retina. In accordance, a number of novel targets are being investigated for their feasibility as DR treatments, including the angiopoietin family of proteins, NSAIDs, immunosuppressants, and oxidative stress inhibitors. Further, a multitude of preclinical research suggests that gene therapy could be a promising therapeutic strategy for the future and negate the need for frequent administration and short-lasting therapeutic effect that currently hinders clinical practice.

## 9. Expert opinion

Although great strides have been made in diagnosing and treating DR, further progress is needed. Future strategies will center on personalized risk stratification, more timely and cost-effective detection of retinopathy through population-based screening and enhanced treatment options, including anti-angiogenic and anti-inflammatory therapies, as well as the development of retino-protective therapies to prevent or delay retinopathy progression and regenerative therapies to repair or replace damaged retinal vessels and retinal neurons.

The clinical significance of each of the biochemical and molecular pathways in DR patients is still unclear. For instance, attempts to inhibit the AGE and polyol pathways in humans have proven unsuccessful, suggesting that their importance in the pathophysiology of DR may be limited. Furthermore, our understanding of the importance of vascular and neuronal cell degeneration needs further refinement given the lack of consensus in our explanation of the relationship between the vascular pathophysiology of DR (hyperpermeability and angiogenesis) and retinal degeneration. In particular, we feel that more research is required to determine whether vascular deterioration potentiates neuronal dysfunction or vice versa; however, we are hopeful that this question will be answered over the next decade or so with multiple groups now using fundus photography and electroretinograms to address the issue.

We are excited by recent findings that suggest the traditional type I and type II DM model is overly simplistic, and there are in fact five subtypes of the condition. Over the coming years, we hope that our field seeks to understand the relevance of this finding to DR, and efforts are made to identify similar subgroups of DR patients. We feel the field has been slow to realize the potential of personalized medicine, and we hope that the identification and characterization of specific genotypic and phenotypic biomarkers facilitates the introduction of targeted therapeutics.

Regarding DR therapeutic paradigms, a number of therapies are now available to clinicians and these have demonstrated modest benefits in patients. While VEGF plays a central role in retinal neovascularization and vascular hyperpermeability in diabetes, it is only one of many angiogenic factors that are upregulated in DR. Future anti-angiogenic therapies for DR are likely to be individualized and will typically target more than one factor. An example is the bispecific monoclonal antibody against Ang-2 and VEGF that is currently undergoing clinical evaluation in Phase III trials (Genentech/Roche). In addition, increasing awareness of retinal neuronal injury in diabetes and the interplay between neurons, glia, and blood vessels (the *NVU*) has focused attention on neuroprotective therapies. The administration of therapies to minimize glutamate-induced excitotoxicity and neuronal apoptosis as well as regulation of RGC apoptosis are currently under evaluation in DR and may be used in future.

Improvements in the treatment of DR and DME will go hand-in-hand with improved treatment of diabetes. The importance of this cannot be understated given the profound influence of glycemic control on the risk or DR and the rate of its progression. Finally, regenerative therapies may have a role to play in DR. Endothelial progenitor cells are endogenous stem-like cells that ordinarily serve roles in vascular repair, including in the retina. Work is underway to identify the most appropriate endothelial cell type for retinal vascular repair and to refine methods for the production of clinical-grade progenitor cells at scale.

In particular, we are excited by recent reports detailing the efficacy of gene therapies for DR. These drugs represent an opportunity to improve patient outcomes in terms of reducing the need for frequent ocular injections and providing superior efficacy through constant therapeutic coverage and targeted delivery to the disease site. No clinical outcomes have been reported almost 15 years since the first studies demonstrating the promise of gene therapy for DR were published however. Therefore, in order to facilitate the translation of these innovations from ‘bench to bedside,’ improvements in regulatory frameworks and scale-up manufacturing processes are needed; yet, we are confident that these developments will occur over the coming years.

In summary, significant progress has been made toward our understanding of DR over the past few decades. In the future, we hope that improvements in our knowledge of the condition facilitate therapeutic innovations that continue to address unmet medical need and improve patient outcomes, with a focus on the development of targeted medicines.

## Article highlights

- Diabetic retinopathy is a complex disorder, in which the dysregulation of a number of biochemical and molecular signaling pathways is driven by oxidative stress.
- A number of treatment options are available for clinicians, including photocoagulation, glycemic control and anti-VEGF agents.
- These strategies demonstrate modest clinical benefits, but none is yet to fully attenuate disease progression or reverse damage to the retina.
- This has driven investigations into novel targets for the condition, including NSAIDs, immunosuppressants and oxidative stress inhibitors.
- Further, gene therapy has demonstrated that it could provide superior patient outcomes in light of the significant advantages inherent to this therapeutic platform.

This box summarizes key points contained in the article.
